# Path Planning Algorithm for Multi-Locomotion Robot Based on Multi-Objective Genetic Algorithm with Elitist Strategy

**DOI:** 10.3390/mi13040616

**Published:** 2022-04-14

**Authors:** Chong Liu, Aizun Liu, Ruchao Wang, Haibin Zhao, Zhiguo Lu

**Affiliations:** School of Mechanical Engineering and Automation, Northeastern University, Shenyang 110819, China; congliu@me.neu.edu.cn (C.L.); 2110099@stu.neu.edu.cn (R.W.); hbzhao@mail.neu.edu.cn (H.Z.); zglu@me.neu.edu.cn (Z.L.)

**Keywords:** multi-locomotion robot, path planning, genetic algorithm, elitist strategy, multi-objective optimization

## Abstract

The multi-locomotion robot (MLR), including bionic insect microrobot, bionic animal robot and so on, should choose different locomotion modes according to the obstacles it faces. However, under different locomotion modes, the power consumption, moving speed, and falling risk of MLR are different, and in most cases, they are mutually exclusive. This paper proposes a path planning algorithm for MLR based on a multi-objective genetic algorithm with elitist strategy (MLRMOEGA), which has four optimization objectives: power consumption, time consumption, path falling risk, and path smoothness. We propose two operators: a map analysis operator and a population diversity expansion operator, to improve the global search ability of the algorithm and solve the problem so that it is easy to fall into the local optimal solution. We conduct simulations on MATLAB, and the results show that the proposed algorithm can effectively optimize the objective function value compared with the traditional genetic algorithm under the equal weight of the four optimization objectives, and, under alternative weights, the proposed algorithm can effectively generate the corresponding path of the decision maker’s intention under the weight of preference. Compared with the traditional genetic algorithm, the global search ability is improved effectively.

## 1. Introduction

Inspired by bionics, many bionic robots have been developed, including bionic insect microrobot and medical microrobot, bionic humanoid robot, and so on. These types of robots usually have multiple modes of locomotion called multi-locomotion robot (MLR). This kind of robot has a broad prospect in a special work environment, such as pipeline maintenance, drugs transportation inside of human body, terrain exploration, and so on. The authors of [1] designed a humanoid robot system which supports bipedal or quadrupedal walking, climbing, brachiation, and even flying. The algorithm proposed in this paper is mainly based on this kind of robot, but the idea of this algorithm is also applicable to other kinds of MLRs. In the related studies, researchers realized bipedal walking on the flat ground [2,3], quadruped walking on the slope [4], swinging on the ladder [5,6,7], and climbing a vertical ladder [8]. In [2], the researchers proposed the 3-D biped dynamic walking algorithm based on the PDAC, and validated the performance and the energy efficiency of the proposed algorithm. In [4], the researchers determined an optimal structure for a quadruped robot to minimize the robot’s joint torque sum. In [5], the researchers presented a control method to realize smooth continuous brachiation. The authors of [6] proposed an energy-based control method to improve the stability of continuous brachiation. The authors of [7] designed a type of brachiation robot with three links and two grippers, and designed a control method based on sliding-mode control, which improved the robustness and swing efficiency of the robot. In [8], the researchers introduced a vertical ladder climbing algorithm of the MLR only by the posture control without any external sensors.

**Figure 1:**
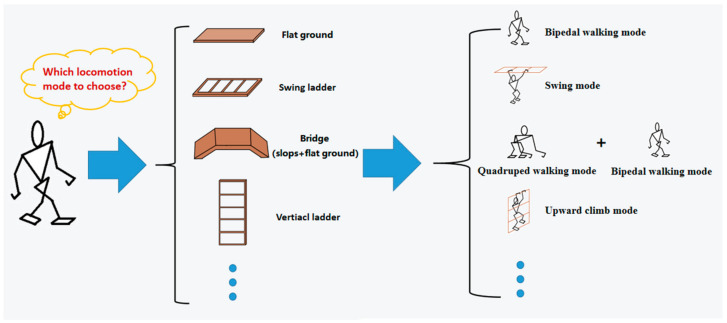
Different locomotion modes for different obstacles.

With the diversification of robot locomotion mode, the application environment of robots has also expanded from the laboratory to the field environment. Due to its high adaptability to the environment, the MLR should choose different locomotion modes according to the obstacles it faces, as shown in Figure 1.

**Figure 2:**
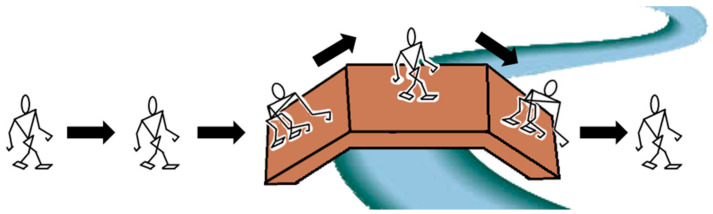
MLR goes over a bridge above a river.

So, multi-locomotion robots can pass obstacles that single locomotion robots could not pass safely in the past by switching locomotion modes. For example, as shown in Figure 2, there is a little bridge above a river, the MLR will choose to switch its locomotion mode from bipedal walking to quadrupedal walking, to go on the slope, and go through the bridge with minimal probability of falling down. For a normal biped robot, walking on the slope of the small bridge in the bipedal way, there will be a high probability of falling down.

Therefore, in the same field environment, the number of possible paths for MLR is greatly increased compared with robots with single locomotion mode. How to choose among many possible paths is a problem to be solved.

**Figure 3:**
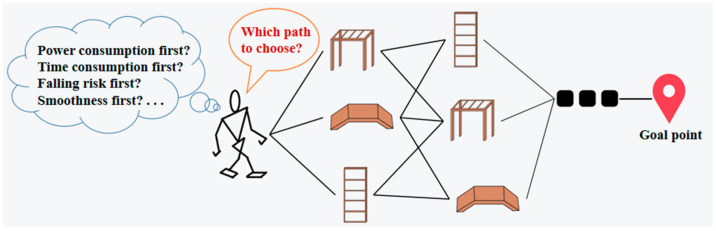
Multi-objective path planning.

Different locomotion modes, such as bipedal and quadrupedal walking, have specific and different capabilities because a robot’s mobility is constrained by the physical and structural conditions for a motion [9]. For example, bipedal walking means low power consumption but is prone to falling down according to [2,10,11]; so, it is suitable to go over flat ground; quadrupedal walking is suitable to climb a slope because of its high stability, but its speed is rather slow [4,12]. Thus, it is necessary to take power consumption, time consumption, and falling risk all into consideration in path planning, as shown in Figure 3. These three goals are normally mutually exclusive; so, the path planning problem is a multi-objective optimization problem.

Many researchers have proposed path planning algorithms for multi-objective optimization, including multi-objective path planning based on the genetic algorithm [13,14], multi-objective path planning based on the particle-swarm optimization algorithm (PSO) [15,16,17], and multi-objective ant colony algorithm path planning [18].

In [19], inspired by the frogs’ behavior, the researchers proposed a multi-objective shuffled frog-leaping path planning algorithm. This algorithm has three objectives: path safety, path length, and path smoothness. In this research, the optimization method of path safety was realized by ensuring the distance between path and obstacle. When the path generated by the algorithm passes through the obstacle, the path safety operator will look for two candidate regions in the vertical direction of this section of the path crossing the obstacle, and determines the new path node according to the vertical distance between the candidate regions and this section of the path in order to generate two new path sections, to avoid passing through the obstacle.

In [20], in order to solve the problem of multi-objective optimization in the global path planning of autonomous intelligent patrol vehicle, which is the shortest path length and the smallest total angle change in the path, the researchers proposed a path planning method based on a multi-objective Cauchy mutation cat-swarm optimization algorithm. The multi-objective problem proposed in this algorithm only considered the path length and path smoothness; thus, the practicability is limited.

In [21], the researchers proposed an improved ant colony optimization-based algorithm for user-centric multi-objective path planning for ubiquitous environments. This algorithm uses the ant colony algorithm to plan a path for vehicle navigation in an urban map considering length, traffic condition, and power consumption; the traffic condition in this research is very similar to the path safety, which provides a reference for considering the path safety in our research.

In [22], the researchers proposed an aging-based ant colony optimization algorithm. The researchers introduced a modification based on the age of the ant into the standard ant colony optimization. This algorithm can find the shortest and the most free-collision path under static or dynamic environment, and, when compared with other traditional algorithms, it proves its superiority. However, path safety is not considered in this algorithm.

In [23], the researchers proposed a multi-objective path planning algorithm based on an adaptive genetic algorithm. In this algorithm, the self-defined genetic operator is used to realize the optimization of the path length and smoothness, and the artificial potential field theory is introduced to realize the planning of the path safety which inspired our research in the optimization of path safety. However, the path safety mentioned in this algorithm only considers the distance between the robot and the impassable obstacle, and does not involve road conditions and the locomotion mode of the robot.

In [24], the researchers proposed a multi-objective genetic algorithm path planning method for reconfigurable robots. This kind of robots can provide high dexterity and complex actions by reconfiguring its shape. This method proposed four objective functions: goal reachability, time consumption, path smoothness, and path safety. Even the reconfigurable robots can provide different shapes to move, but the path safety considered in this method only considered the distance between robots and obstacles rather than considering the falling risk of a robot in different shapes.

In [25], the researchers proposed a multi-objective path planning algorithm for mobile robot, this algorithm has three objectives: length, smoothness and safety. The applicable environment of this algorithm is relatively simple, the environment was only divided into passable and impassable, and the safety optimization only considers the distance between the robot and the obstacles.

In [26], the researchers proposed a path planning method based on interval multi-objective PSO; this method concentrates on three objectives: path time, path safety, and path length. In the optimization of path safety, this method takes the road condition into consideration, which has enlightening values to our works, but the applied objects of these methods are traditional wheeled robots; so, this method is not suitable for MLR.

Based on the path planning research described above, and taking the high adaptability to the complex environment of MLR into consideration, a path planning algorithm for multi-locomotion robot (MLR) based on multi-objective genetic algorithm with elitist strategy (MLRMOEGA) is proposed in this paper. The algorithm considers the power consumption, moving speed, and falling risk of the MLR in different locomotion modes and different environment, and proposes four optimization objective functions: power consumption, time consumption, path falling risk, and path smoothness. Compared with previous studies, optimal safety mostly refers to the distance between the robot and the obstacle. This paper proposes the concept of robot global path falling risk, that is, when the MLR is moving through alternative locomotion modes, there will be a certain falling risk, so there will be a falling risk of each possible path. We calculate the falling risk of each path, and take it into the multi-objective problem to be considered. To solve the problem of premature convergence of the Genetic Algorithm, we propose two operators: a map analysis operator and a population diversity expansion operator, to improve the population diversity in the algorithm process.

The rest of this paper is organized as follows: Section 2 is the introduction of the environment building method and the problem statement. Section 3 is the introduction of the Genetic Algorithm and the proposed algorithm. Section 4 is the implementation of the multi-objective path planning including building global environment and details of the algorithm. Section 5 is simulation experiment. Section 6 is the conclusion.

## 2. Problem Statement and Preliminaries

### 2.1. Method of Building the Global Path Planning Environment

The path planning of a robot is divided into two steps: first, abstract out a global map containing real environment information, then, execute the path planning algorithm and the path will be generated.

**Figure 4:**
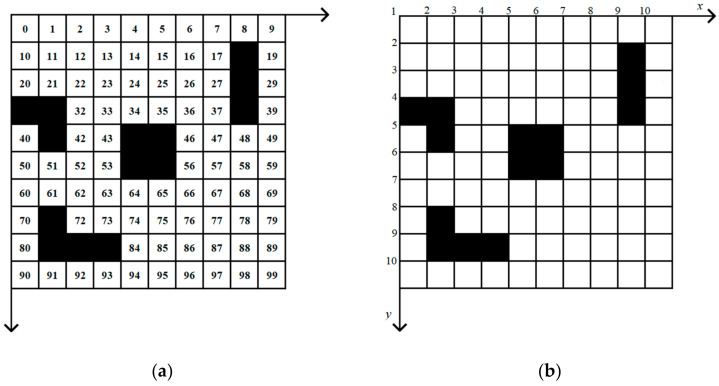
Example of a 10 × 10 grid map: (**a**) index to the grid; (**b**) coordinate of the grid.

In this paper, we map the environment on a grid of size *n* × *n*, where the grid size *n* is decided depending on the accuracy required in the path planning problem [27]; the bigger *n* is, the more accurate the map will be. Each grid of the map contains environmental information of its location. There is an example of a 10 × 10 grid map, as shown in Figure 4, in this example, black grids represent impassable obstacles, white grids are traversable. The position of each grid can be obtained by index or coordinate values, which can be converted to each other as Equation (1):

(1) $$\;\;\;\;\;\;ind=A\times y+x-\left(A+1\right)\;\left\{\begin{array}{l} \;\;y=\frac{ind}{A}+1\\ x=ind\%A+1 \end{array}\right.$$

In this equation, *ind* is the index value of gird, *A* is the size of the grid map, ⌊⌋ means to round down the value to the closest integer, % means to take the remainder as the result.

### 2.2. Optimization Objective Functions

This paper proposes four optimization objectives for MLR: power consumption, time consumption, path falling risk, and path smoothness.

One path consists of many grid cells from the grid map and we call them nodes of paths, and we divide one path into several sections. We call them path sections based on the locomotion modes that the MLR takes. *d_i_* is the Euclidean distance of each path section as shown in Equation (2) [23]:

(2) $$d_{i}=\sum_{j=1}^{J}\sqrt{{\left(x_{j}-x_{j-1}\right)}^{2}+{\left(y_{j}-y_{j-1}\right)}^{2}}\;\;\;\;\;\;1\leq x,y\leq A$$

In this equation, *i* is the number of this path section; *j* is the node number in this section; *J* is the total number of nodes in this section; *A* is the size of the grid map.

1. Power consumption: In order to pass different road conditions, the MLR will choose alternative locomotion modes with different power consumption. In the real environment, the energy stored by the MLR itself is very limited, which greatly restricts the activity duration of the MLR. Therefore, it is very necessary to include power consumption into the optimization objective in path planning. The power consumption is calculated by Equation (3):

(3) $$f_{1}\left(\vec{p}\right)=\sum_{i=1}^{N}C_{i}•d_{i}$$

In this equation, $\vec{p}$ is a vector that consists of the nodes’ coordinate of one path; *C_i_* is the power consumption of different path sections with different locomotion modes; *N* is the total path section count of this path.

2. Time consumption: Different locomotion modes bring different time consumption. We expect MLR to perform time-sensitive tasks, such as terrain detection, material transport, rescue and so on; so, time consumption must also be considered in path planning. It is calculated by Equation (4):

(4) $$f_{2}\left(\vec{p}\right)=\sum_{i=1}^{N}\frac{d_{i}}{S_{i}}$$

*S_i_* represents the moving speed of the MLR in this path section with one locomotion mode; *i*, *N*, and $\vec{p}$ represent the same quantities as in Equation (3).

**Figure 5:**
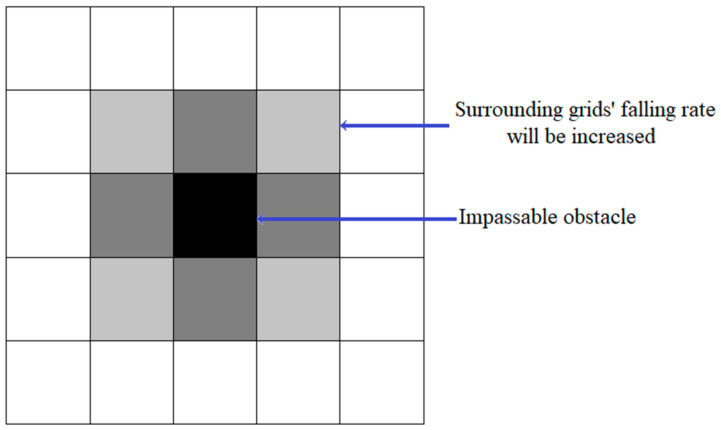
The diffusion of artificial potential field.

3. Path falling risk: Different locomotion modes not only affect the power consumption and time consumption of one path, but also the falling risk. Compared with ordinary wheeled mobile robots, the locomotion modes of MLR have higher risk of falling, and its design is aimed at a more complex field environment; so, it is also important to ensure that the falling risk of MLR in the process of moving is within an acceptable range. In addition to considering the falling risk of the MLR in different locomotion modes, the condition when the MLR is too close to an impassable obstacle should also be taken into consideration; so, we refer to the theory of artificial potential field [28] and transform the diffusion of the potential field into the influence on the falling risk of surrounding girds. When one grid in the map represents an impassable obstacle, the falling risk of the eight surrounding grids will be increased, as shown in Figure 5.

We define the influence of impassable obstacles on the surrounding grid as shown in Equation (5):

(5) $$\;\left\{\begin{array}{l} \;P_{\mathrm{sg}}\left(f\right)=P_{\mathrm{raw}}\left(f\right)+0.05\begin{array}{c} \;\;D=1 \end{array}\\ P_{\mathrm{sg}}\left(f\right)=P_{\mathrm{raw}}\left(f\right)+0.03\begin{array}{c} \;\;D=\sqrt{2} \end{array} \end{array}\right.\;D=\sqrt{{\left(x_{\mathrm{ip}}-x_{\mathrm{sg}}\right)}^{2}+{\left(y_{\mathrm{ip}}-y_{\mathrm{sg}}\right)}^{2}}$$

In this Equation, $P_{\mathrm{sg}}\left(f\right)$ represents the falling risk of surrounding girds of the impassable obstacle; $P_{\mathrm{raw}}\left(f\right)$ represents the falling risk of the grids when there is no impassable obstacle around; *D* is the Euclidean distance between the impassable obstacle and the surrounding grids; *x*_ip_, *y*_ip_ are the coordinates of the impassable obstacle; *x*_sg_, *y*_sg_ are the coordinates of the surrounding girds.

We assume that whether the MLR falls or not is independent of the path at any different nodes and we calculate the falling risk with Equation (6):

(6) $$f_{3}\left(\vec{p}\right)=1-\prod_{k=1}^{n}\left[1-P_{k}\left(f\right)\right]$$

*k* is the count of node in an individual. *n* is the total node count in an individual. *P_k_*(f) is the falling risk when the MLR passes one node.

4. Path smoothness: We want the path generated by the algorithm to be as smooth as possible, and we define the sum of all the angles in the path as the smoothness of that path. The smoothness of a path is calculated by Equation (7) [23]:

(7) $$f_{4}\left(\vec{p}\right)=\sum_{t=1}^{T}\begin{array}{c} \left(\pi -\arccos\left(\frac{\left(b^{2}+c^{2}-a^{2}\right)}{2bc}\right)\right) \end{array}$$

In this Equation: $a=\sqrt{{\left(x_{k+1}-x_{k-1}\right)}^{2}+{\left(y_{k+1}-y_{k-1}\right)}^{2}}$, $b=\sqrt{{\left(x_{k}-x_{k-1}\right)}^{2}+{\left(y_{k}-y_{k-1}\right)}^{2}}$, $c=\sqrt{{\left(x_{k+1}-x_{k}\right)}^{2}+{\left(y_{k+1}-y_{k}\right)}^{2}}$, *k* is the count of node in one path, *T* is the total number of turns in one path.

5. The synthetic objective function: We synthesize the above four objective functions and linearly weighted them as the final objective function. As shown in Equation (8) [23]:

(8)  f(p→)=cw×f1(p→)+tw×f2(p→)+rw×f3(p→)+aw×f4(p→)cw+tw+rw+aw=1  cw≥0, tw≥0, rw≥0, aw≥0

In this equation, *cw* is the power consumption weight, *tw* is the time consumption weight, *rw* is the falling risk weight, and *aw* is the smoothness weight. The weight values are determined by decision makers through experience or practical requirements; they represent the importance that decision-makers attach to different objective functions. The higher a weight value is, the more attention it receives in multi-objective optimization problems.

## 3. Introduction of MLRMOEGA

### 3.1. Overview of Genetic Algorithm

Genetic Algorithm (GA) was proposed by Professor J. Holland in 1975. This algorithm simulates the genetic processes in nature, that is, starting from an initial population, through selection, crossover and mutation operation, a new population with higher adaptability to the environment can be obtained. In theory, the population will keep approaching to a better search space and, finally, will become a group of individuals which is most adaptable to the environment.

In our research, we use grid map to describe the environment; with this method, the path planning problem becomes a discrete problem and the GA has a good performance in dealing with discrete problems. In the process of GA, each grid can play the role of genes very well, and the path composed of grids is a chromosome. Combining the grid number of each path and the environmental information contained in each grid, the power consumption, time consumption, path falling risk, and path smoothness of each path can be clearly obtained, and then, the fitness of each path can be obtained. We explain this in Section 4.1.

There are other intelligent algorithms, such as ant colony algorithm (ACO) and particle swarm optimization (PSO). ACO works efficiently in graph and network space, such as the traveling salesman problem (TSP) and the scheduling problem [21], but is unsuitable to solve path planning problems in a grid map. The original POS has been designed to work in continuous space, and it needs some major changes to adapt to path planning problems [21]. As we use a grid map to describe the environment in our research, it turns out that POS is not very suitable.

**Figure 6:**
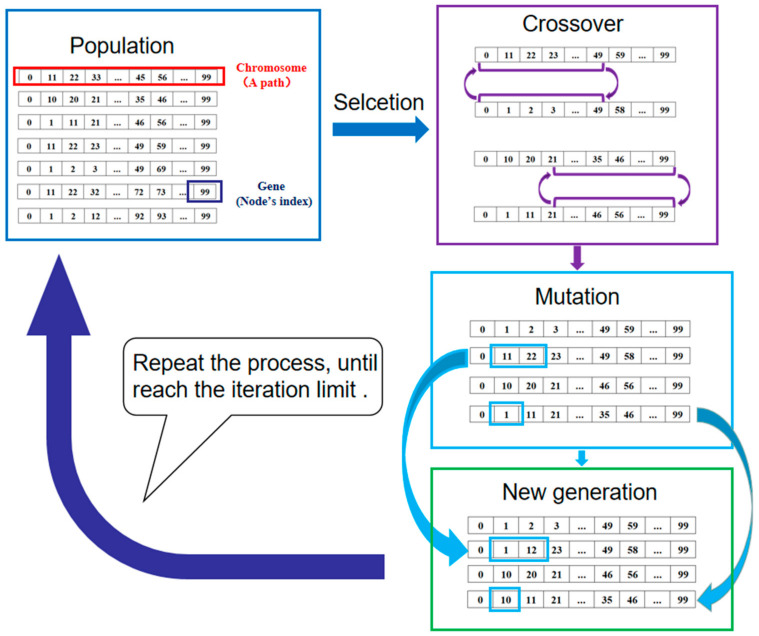
GA used in path planning.

The application of GA in path planning is shown in Figure 6.

### 3.2. Elitist Strategy

**Figure 7:**
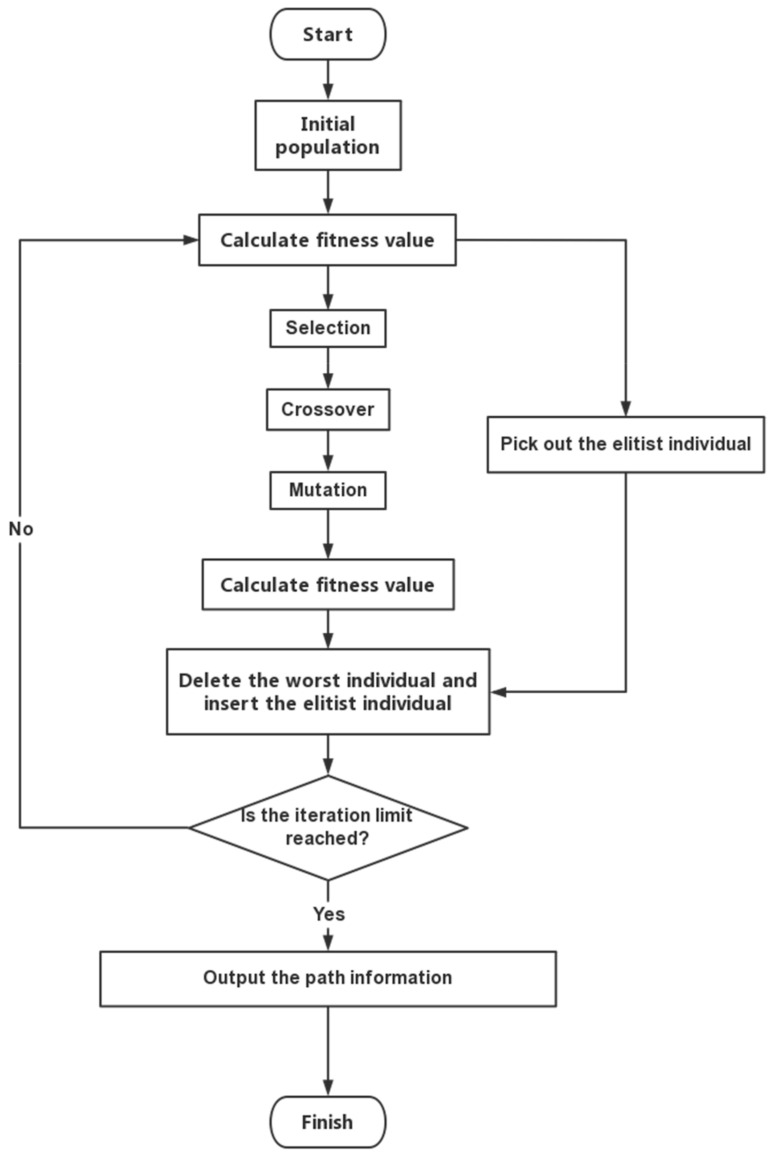
Flowchart of EGA.

When GA only includes selection, crossover, and mutation, it is called Simple Genetic Algorithm (SGA). It is proved that the SGA does not converge almost surely to the set of populations that contains the optimum point as one of its points [29]; so, we introduce the Elitist Strategy into SGA. It is shown that Elitist Strategy helps in achieving better convergence in SGA [30]. The Simple Genetic Algorithm with Elitist Strategy is called EGA, the flowchart of EGA is shown in Figure 7.

Elitist strategy improves the global convergence of GA, but on the other hand, it also makes a local optimal individual not easy to be eliminated and continues to be retained in the genetic processes, thus affecting the global search ability of algorithm, and eventually leads to premature convergence; so, it is usually used in conjunction with other operators. In the following section, we propose two operators to work with EGA, in order to improve the global search ability of the algorithm.

### 3.3. The Proposed Algorithm

**Figure 8:**
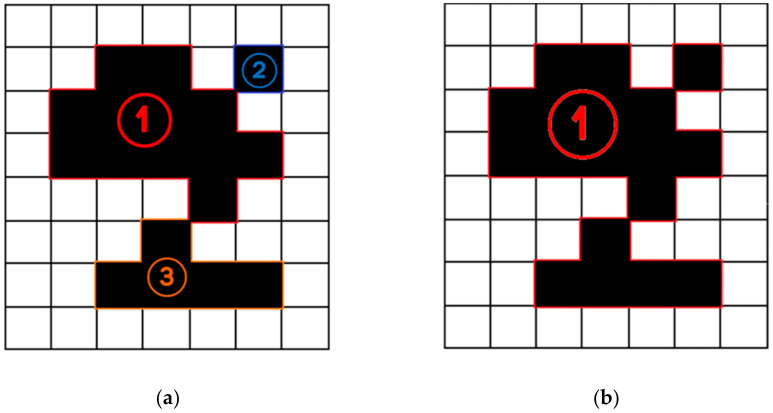
(**a**) Four-connected principle; (**b**) eight-connected principle.

The premature convergence is generally due to the loss of diversity within the population [31]. In order to solve this problem, the map analysis operator and the population diversity expansion operator are proposed in this paper:

- Map Analysis Operator: Once the algorithm starts, the map analysis operator will analyze the grid map, divide various obstacles in the grid map into regions and store the index values of each region, respectively. There are two methods to divide a map into regions: the four-connected principle and the eight-connected principle. We assume the current grid coordinate is (*x*, *y*), the four-connect principle considers the four grids with (*x* + 1, *y*), (*x* − 1, *y*), (*x*, *y* + 1) and (*x*, *y* − 1) as the same region, and the eight-connect principle considers the eight grids with (*x* + 1, *y*), (*x* − 1, *y*), (*x*, *y* + 1), (*x*, *y* − 1) (*x* + 1, *y* + 1), (*x* − 1, *y* − 1), (*x* − 1, *y* + 1) and (*x* + 1, *y* − 1) as the same region. For example, as shown in Figure 8, the four-connected principle divides this obstacle into three regions as shown in Figure 8a marked by circled numbers, while the eight-connected principle into one region, as shown in Figure 8b. The MLR has eight moving directions in the map; therefore, it is more reasonable to choose the eight-connection principle.

The pseudo-code for the Map Analysis Operator is as follows:
**Map Analysis Operator:****Input:** A matrix map of gray values1: Store all slope grids’ index in the matrix map of gray values2: Store all non-slope grids’ index in the matrix map of gray values3: Set the gray values of all non-slope grids to 0 in the matrix map4: Set the gray values of all slope grids to 1 in the matrix map, get a new matrix map5: Divide the new matrix map into regions according to the eight-connected principle6: The indexes of the slope grid are stored according to the divided regions and saved into a cell array//Other kinds of obstacles are divided into regions in the same way7: Combine all grids’ index stored according to the divided regions into one cell array**Output:** A cell array contains the grids’ index according to the divided regions

- Population Diversity Expansion Operator: In the genetic process, in order to improve the global search ability of GA, the most direct method is to improve population diversity. For MLR, the diversity of the population is reflected in whether the locomotion modes adopted by MLR are sufficiently diverse, that is, whether the path individuals in the population have passed the obstacles that MLR needs to adopt alternative locomotion modes to pass in the global map. Therefore, we use the population diversity operator to check whether all the regions obtained by the map analysis operator have been gone through by the path in the population. If some regions have not passed through, the paths going through those regions will be generated to improve the population diversity.

The pseudo-code for the Population Diversity Expansion Operator is as follows:
**Population Diversity Expansion Operator:****Input:** The divided regions cell array from map analysis operator; the cell array composed of populations of this generation1: All path individuals in the population are intersected with the region in the divide regions cell array2: Take the region that has no intersection with any individual in the population, this is the unpassed region3: A random point in the unpassed region is selected to generate a new path individual4: Put the new path individual into the population**Output:** The population after diversity expansion

**Figure 9:**
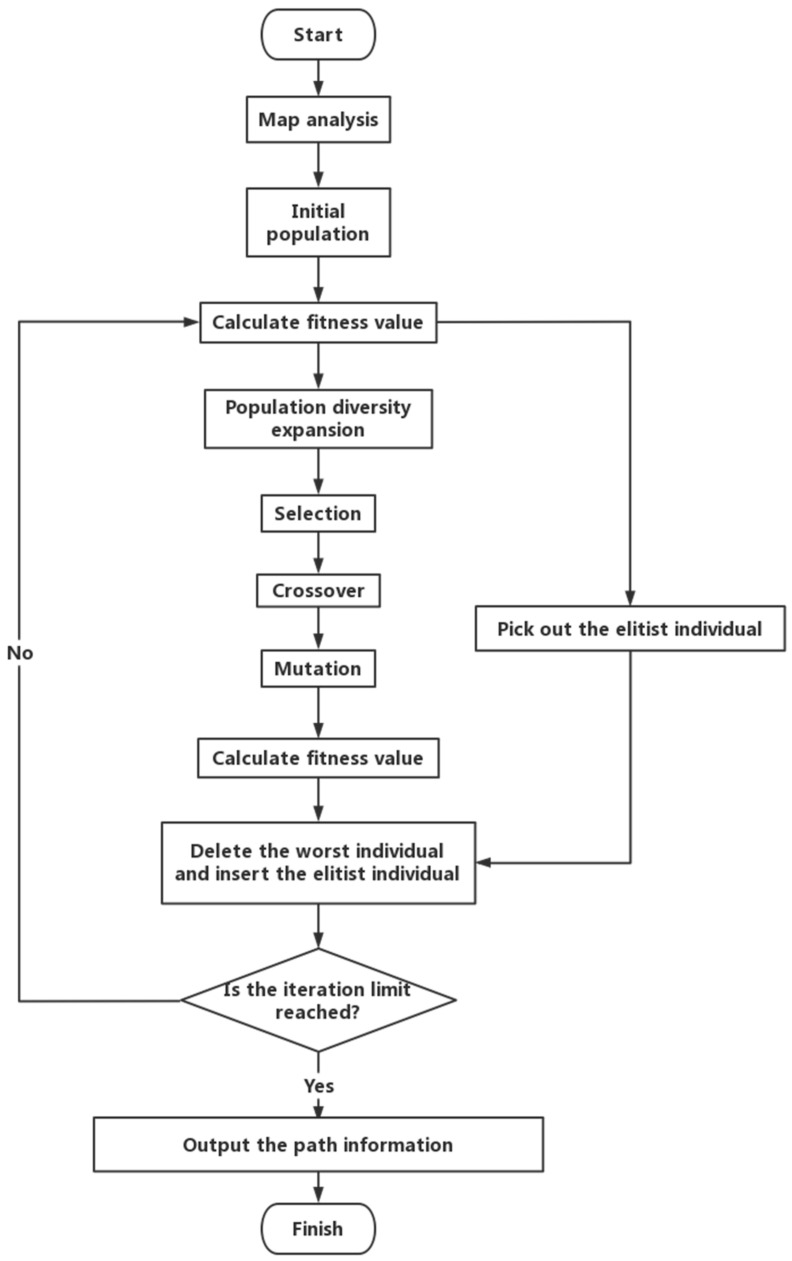
Flowchart of MLRMOEGA.

The flowchart of MLRMOEGA is shown in Figure 9.

## 4. Implementation

### 4.1. Encoding Method

One of the main factors in the implementation of SGA is the encoding method. In conventional GA, solutions are encoded in binary strings, however, several different encodings are also possible depending on the problems [32], and we use floating point encoding in this paper. In this method, each gene value of the individual is represented by a real number in a certain range, and the encoding length is equal to the number of decision variables. The encoded individual is a set of grid index, representing a possible path, as shown in Equation (9).

(9) $$\vec{p}=\left\{index_{1},index_{2},\ldots ,index_{i},\ldots ,index_{n}\right\}$$

**Figure 10:**
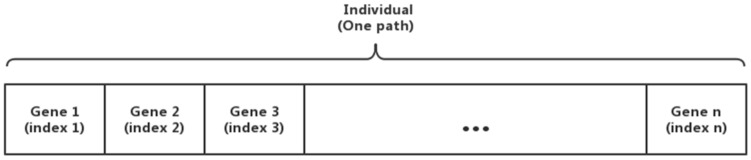
Composition of one individual.

The composition of one individual in our research is shown in Figure 10.

### 4.2. The Evaluation Function

The evaluation function is used to evaluate the quality of an individual in the algorithm process. In the selection operation, the larger the value of the evaluation function is, the more likely it is to be retained in the next generation.

1. The power consumption evaluation function: To make the data dimensionless, we take the natural logarithm of the data, and use the same method to calculate the other data. According to Equation (3), the evaluation function of power consumption is Equation (10):

(10) $$F_{1}({\vec{p}}_{pl})={\left(ln(f_{1})\right)}^{-1}$$

2. The time consumption evaluation function: According to Equation (4), the evaluation function of time consumption is Equation (11):

(11) $$F_{2}({\vec{p}}_{pl})={\left(ln(f_{2})\right)}^{-1}$$

3. The path falling risk evaluation function: According to Equation (6) and to make the data dimensionless, we have the evaluation function of falling risk as shown in Equation (12):

(12) $$F_{3}({\vec{p}}_{pl})={\left(ln\left(f_{3}\times 1000-\mathrm{BC}\right)\right)}^{-1}$$

BC is a constant value related to the motion data of the robot, and its function is to reduce the quantitative difference between the falling risk evaluation function and other evaluation functions.

4. The path smoothness evaluation function: The smoothness evaluation function is quite different from Equation (7). First, we define a variable *α_t_*, it represents the value of the angle formed by every three consecutive nodes as shown in Equation (13):

(13) $$\alpha_{t}=\pi -arccos\left(\frac{\left(b^{2}+c^{2}-a^{2}\right)}{2bc}\right)$$

The variables in this equation represent the same quantities as in Equation (7).

The evaluation function of the smoothness consumption is Equation (14):

(14) F4p→pl=ln5+∑t=1T  10 90°<αt<170°30 45°<αt≤90°90              αt≤45°−1

*T* is the total number of turns in an individual. In Equation (14), each individual has an initial value which is 5, to prevent taking the natural logarithm of 0. We assign a penalty value to every angle that occurs in each individual, when the angle is obtuse, the penalty value is small; then, with the smaller $\alpha_{t}$, there will be a greater penalty value.

### 4.3. Execution Process of MLRMOEGA

(1) Map analysis: According to the eight-connected principle, the obstacles in a grid map are divided into regions.
(2) Initial population: Generate the required number of individuals for the initial population.
(3) Calculate fitness value: Normally, a common method used to solve multi-objective problems is by a linear combination of the objectives, in this way creating a single-objective function to optimize [33]. In is step, we linearly weight the evaluation functions as shown in Equation (15):

(15) F(p→pl)=cw×F1(p→pl)+tw×F2(p→pl)+rw×F3(p→pl)+aw×F4(p→pl)cw+tw+rw+aw=1  cw≥0, tw≥0, rw≥0, aw≥0

The variables in this equation represents the same quantities as in Equation (8).

(4) Pick out the elitist individual: according to the previous step, we pick out the individual with highest $F(\vec{p})$ called elitist individual, and retain it to step (10). This is the first step of the Elitist strategy.
(5) Population diversity expansion: Check population diversity and expand population to improve population diversity when population diversity declines.
(6) Selection: In the selection process, we use the proportion select method. The $F(\vec{p})$ of all the individuals in this generation has been calculated in the previous step, so we sum them up, and the fitness of each individual divided by this sum is the probability of being selected in the proportional selection method for one individual, that is, Equation (16):

(16) $$P\left(A_{pl}\right)=\frac{F({\vec{p}}_{pl})}{\sum_{pl=1}^{PL}F({\vec{p}}_{pl})}$$

In this equation, event A*_pl_* represents the *pl*th individual in the population which is selected into the next generation, *PL* is the population quantity.

(7) Crossover: We set a crossover probability called CP in advance, and then check whether two individuals in the population share one gene, that is, whether they pass through one same node, and if they do, we use the probability to determine whether they do crossover. Once the crossover happened, all the genes that follow the same gene in two individuals are swapped and generate two new individuals.
(8) Mutation: We set a mutation probability called MP in advance, check each individual in this population; if one individual needs to mutate, we delete randomly three genes which are the nodes in this individual, then generate a new individual through program operation.
(9) Calculate fitness value: We calculate individuals’ fitness value again.
(10) Delete the worst individual and insert the elitist individual: This is the second step of the Elitist strategy. According to the previous step, we delete the individual with the lowest $F(\vec{p})$, and put in the elitist individual from step (4).
(11) Check whether the iteration limit is reached: If the iteration limit is reached, output the path information, otherwise, go back to step (3), and continue the iteration.

## 5. Simulation and Analysis

The simulation experiment is conducted by MATALB 2020b, the configuration of computer is Core i5 CPU (3.8 GHz), 8 GB RAM, and Windows10 system. The idea of our experiments in this section is to test the effectiveness of the proposed algorithm in random 20 × 20 grid maps and compare the performance of the proposed algorithm with other algorithms. Then, we test the proposed algorithm performance in a simulated field environment which is abstracted into a 30 × 30 grid map.

### 5.1. The Software System

**Figure 11:**
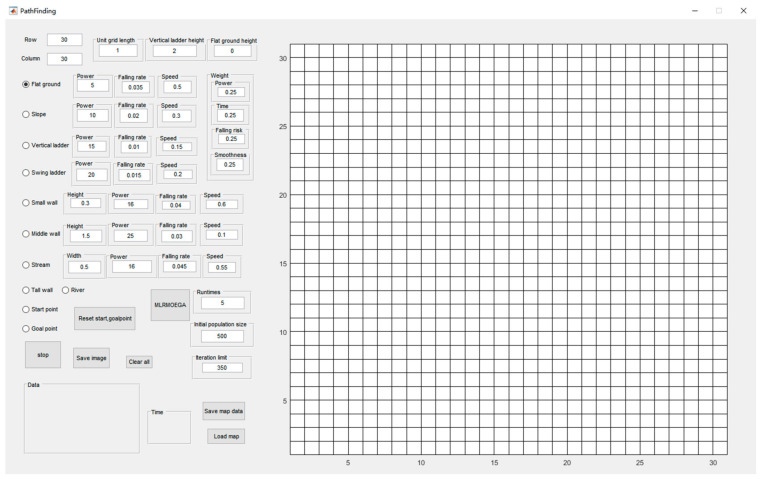
User interface (with 30 × 30 grid map).

To conduct the simulation, we developed a user interface, as shown in Figure 11. In this software, we can input and adjust the data of the MLR moving modes and edit the global map by clicking the grid.

There are 10 kinds of grids and 7 kinds of locomotion modes in total. In addition to the mentioned flat ground, swing ladder, slope and vertical ladder and their corresponding locomotion modes in Section 1, there are six other kinds of grids and other locomotion modes: start and goal point seemed as flat ground, river, and high wall, which are impassable obstacles, stream for long jump mode, a small wall for which the MLR chooses the high jump mode to go through, and a middle wall over which the MLR will climb.

To verify the validity of the algorithm, we assume a set of MLR data as shown in Table 1:

### 5.2. Verify the Validity of Artificial Potential Field Theory

**Figure 12:**
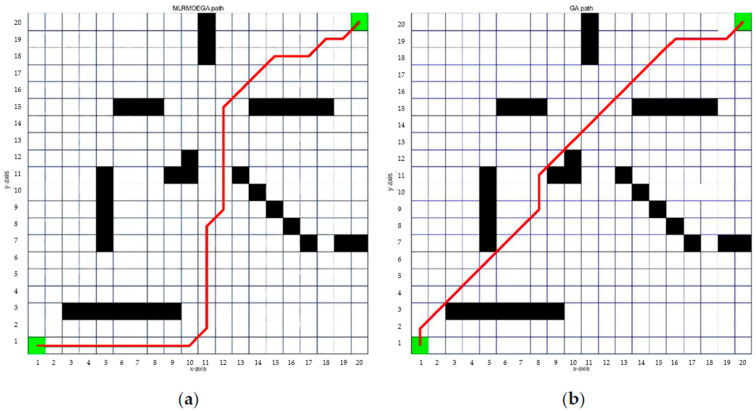
Results of artificial potential field test (with 20 × 20 grid map): (**a**) MLRMOEGA path (Falling risk: 0.7303); (**b**) SGA path (Falling risk: 0.7326).

**Figure 13:**
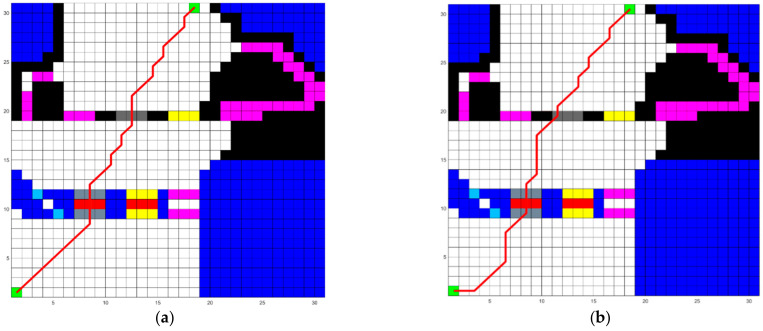
Results of artificial potential field test (with 30 × 30 grid map): (**a**) MLRMOEGA path (Falling risk: 0.70868); (**b**) SGA path (Falling risk: 0.7137).

Since the four objective functions will influence each other in the process of multi-objective optimization, we first verify the effectiveness of the falling risk optimization process with artificial potential field theory. The parameters of SGA and MLRMOEGA are shown in Table 2. We use SGA and MLRMOEGA, respectively, to generate a path 10 times in a random environment (20 × 20 grid map) and a simulate environment (30 × 30 grid map). In the simple environment, there is only one kind of impassable obstacle, randomly placed on a 20 × 20 grid map. The introduction of the simulated environment and the obstacles in it has been given in Section 5.4.3. The results are shown in Table 3 and Table 4, the falling risk of paths generated by MLRMOEGA is significantly lower than the falling risk of paths generated by SGA (*p*-value of data in Table 3 is 0.000395 and *p*-value of data in Table 4 is 0.00286). We pick out the paths with the minimum falling risk generated by the two algorithms, as shown in Figure 12a and Figure 13a, red lines mean the path generated by algorithm, the path generated by MLRMOEGA effectively avoids walking along or getting too close to the impassable obstacles that can generate artificial potential fields which are black blocks shown in figures compared with the path generated by SGA in Figure 12b and Figure 13b.

### 5.3. Effect of Design Parameters on the Proposed Algorithm

In this section, we present the influence of the following design parameters: iteration limit and initial population quantity. The MLR data are shown in Table 1, and other parameters of the algorithm are shown in Table 5. The way to evaluate the influence of design parameters on the algorithm is to calculate the synthetic objective function shown in Equation (8). The smaller the synthetic objective function value is, the better the influence of the design parameters.

The results below are from the 30 × 30 grid map. The more grid cells in the map, the larger the number of the iteration limit and the initial population quantity required by the algorithm; therefore, the design parameters of the algorithm based on the 30 × 30 grid map test are also applicable to the 20 × 20 grid map.

#### 5.3.1. Iteration Limit

**Figure 14:**
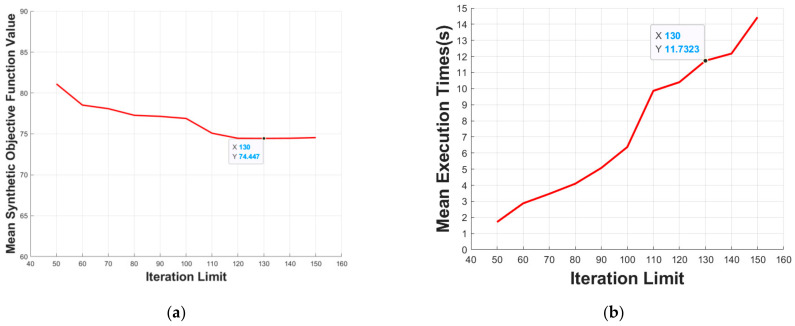
Effect of the iteration limit on: (**a**) mean synthetic objective function value; (**b**) mean execution time.

The initial population quantity is set to 100. By changing the iteration limit of the proposed algorithm from 50 to 150, we execute the proposed algorithm 10 times at each iteration limit, and then take the mean value of the synthetic objective function and execution time. As shown in Figure 14a, the mean synthetic objective function value of the obtained path for testing the environment converged to a small range. In addition, with the increase of the iteration limit, the mean execution time has an upward trend, as shown in Figure 14b. Taking the mean synthetic objective function value and mean execution time into consideration, the value 130 is selected to be the optimal iteration limit of the environment with a 30 × 30 grid.

#### 5.3.2. Initial Population Quantity

**Figure 15:**
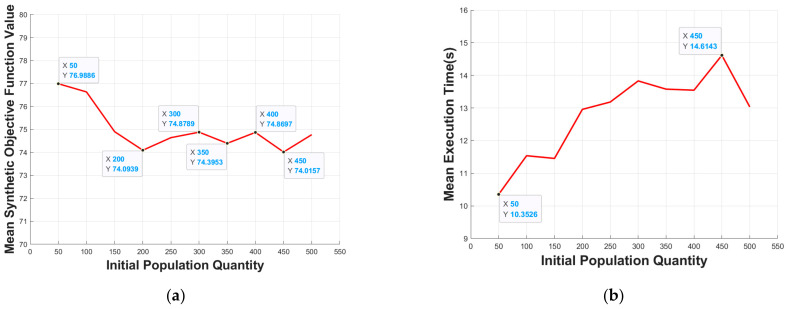
Effect of the initial population quantity on: (**a**) mean synthetic objective function value; (**b**) mean execution time.

The iteration limit is set to 130. We execute the proposed algorithm 10 times at each initial population quantity, and then take the mean value of the synthetic objective function value and execution time. Through experiments, we come to the conclusion that the optimal initial population quantity of environment with a 30 × 30 grid is 200. As shown in Figure 15a, with the increase of the initial population quantity, the search space of the proposed algorithm is expanded, search ability is improved; so, the mean synthetic objective function value decreased, and, after reaching a certain initial population quantity, the mean synthetic objective function value converged to a small range. In Figure 15b, with the increase of initial population quantity, the amount of the data needed to be processed increased. Considering the existence of genetic algorithm contingency, although there are certain fluctuations, the mean execution time still shows a general upward trend.

### 5.4. Test Algorithm Performance

#### 5.4.1. Compared with SGA

We compared the performance of the proposed algorithm with SGA in this section. In the previous part, the optimal design parameters for a 30 × 30 grid map are obtained. With the increase of grid map size, the initial population quantity and iteration limit required by GA to achieve good results also increased; so, the design parameters of the 30 × 30 grid map obtained in our test are also applicable to the 20 × 20 grid map.

**Figure 16:**
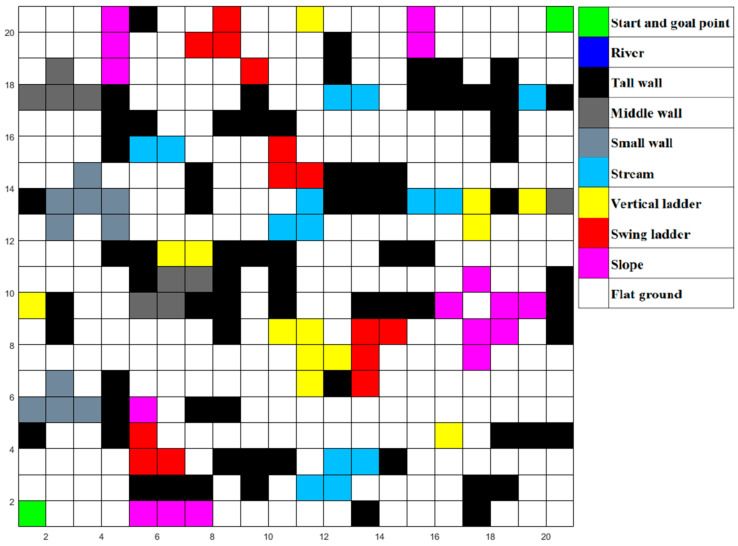
The grid map of the testing environment (20 × 20 grid cells).

The test environment is as shown in Figure 16. This 20 × 20 grid map was generated randomly, we set the proportion of impassable obstacles to 20%, and the proportion of other kinds of passable obstacles to 4%. We generate the map for 7 times; each time we generate the map with flat ground and one kind of obstacle, then, we integrate the 7 maps into one map. In the process of integration, there will inevitably be many obstacles in the same grid; so, we set an obstacle in the coincidence grid on the premise of ensuring that there will not be a large error in obstacle setting proportion.

The design parameters of SGA and MLRMOEGA are shown in Table 6.

**Figure 17:**
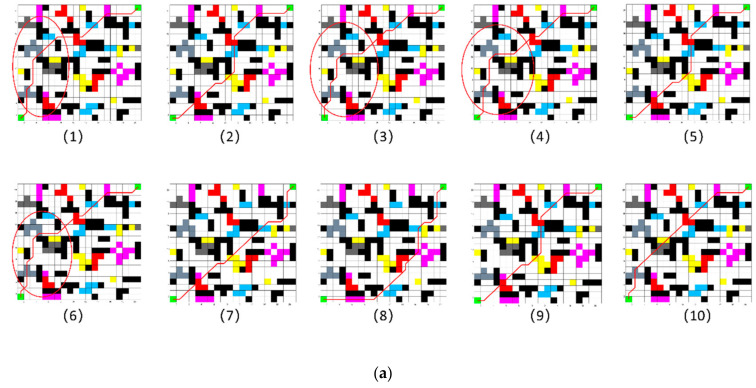

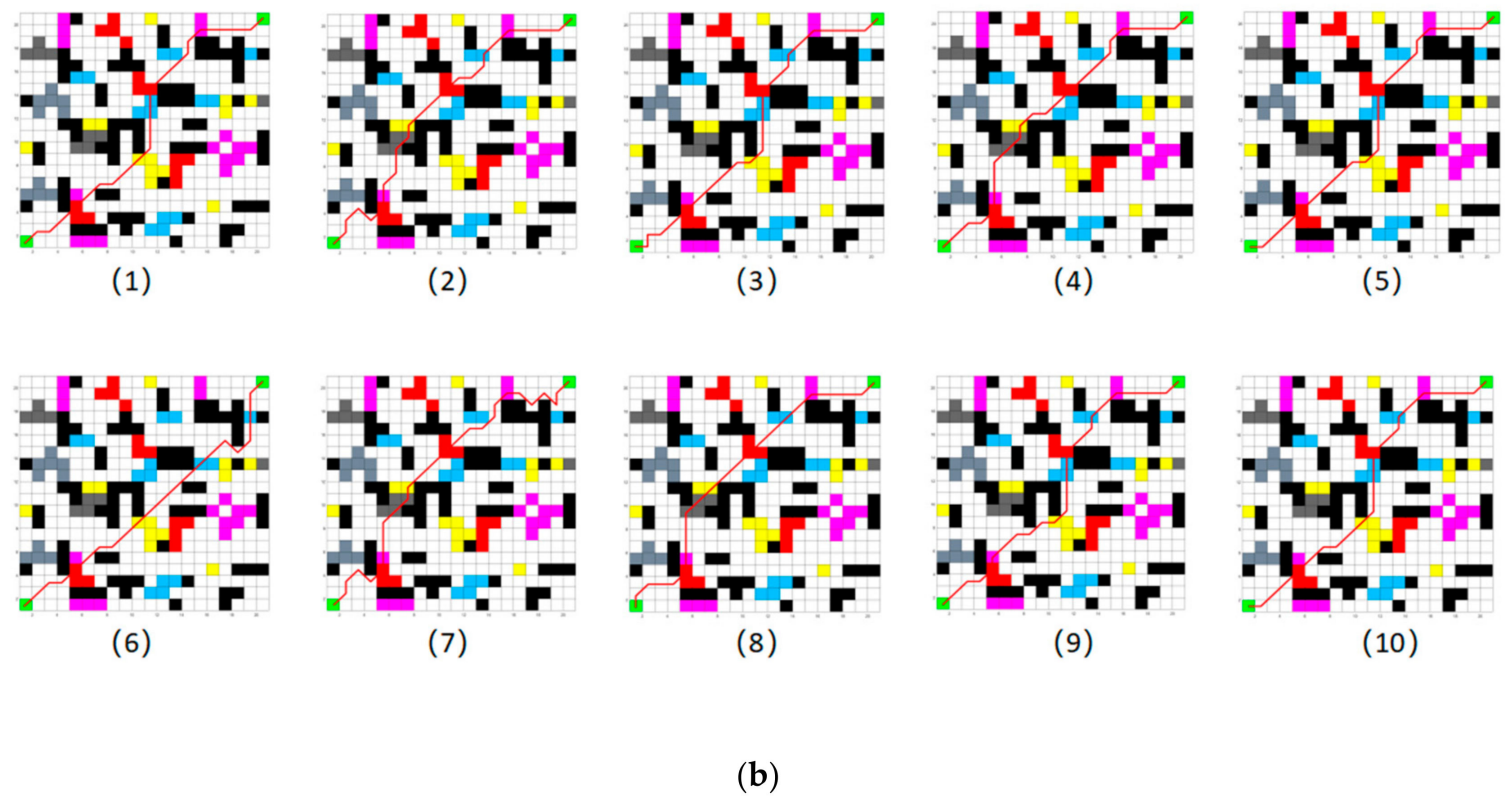
The paths of 20 × 20 grid map generated by: (**a**) MLRMOEGA; (**b**) SGA.

We first use SGA and MLRMOEGA, respectively, to generate a path 10 times in the testing environment shown in Figure 16, and then take the mean value of the synthetic objective function. The synthetic objective function value of SGA paths is calculated by the same way as MLRMOEGA paths’, the results are shown in Table 7. To clarify, Figure 17 shows the paths generated by different algorithms.

Combined with the results in Table 7, it can be seen that the objective function values of the path that passes through the region circled by red in Figure 17a are significantly smaller than other paths. Due to the better global search ability and convergence of MLRMOEGA, after 10 tests, 4 paths pass through the red-circled region and are finally retained. While under the same design parameters, the objective function value of paths generated by SGA are larger (the *p*-value of the runs in Table 7 is 0.0159) and the global search performance of SGA is worse than that of MLRMOEGA.

We also conducted experiments in five other random 20 × 20 grid maps, and the results are shown in Table 8. It can be seen that in different random environments, the paths’ objective function value generated by MLRMOEGA is significantly smaller than that generated by SGA in most cases (the *p*-values of these five tests are 0.05430, 0.002721, 0.000009037, 0.0008754, and 0.009397).

#### 5.4.2. Compared with Multi-Objective A* Algorithm

The A* algorithm is a deterministic search method based on traversal. The original A* algorithm is mainly used to solve the shortest path in global path planning. Due to its simple and intuitive search process, it is a typical algorithm for global path planning problems.

The A* algorithm compares the heuristic function values *F* of the eight neighbor grids of the current grid to determine the next path grid. However, when there are multiple minimum values, the A* algorithm will randomly determine one grid as the next path gird, so it cannot guarantee the optimal path.

We use the proposed MLRMOEGA and compare it with an improved A* algorithm for multi-objective optimization. For this kind of multi-objective, the A* algorithm only has three optimization objectives: power consumption, time consuming, and falling risk, without path smoothness. Therefore, in this test, these two algorithms will not optimize the path smoothness, to ensure even test conditions. The test is performed on random 20 × 20 grid maps, which were generated in the same way as in Section 5.4.1. In order to make the contrast more obvious, we reduced the proportion of various obstacles. We set the proportion of impassable obstacles to 10%, and the proportion of other kinds of passable obstacles to 2%.

**Figure 18:**
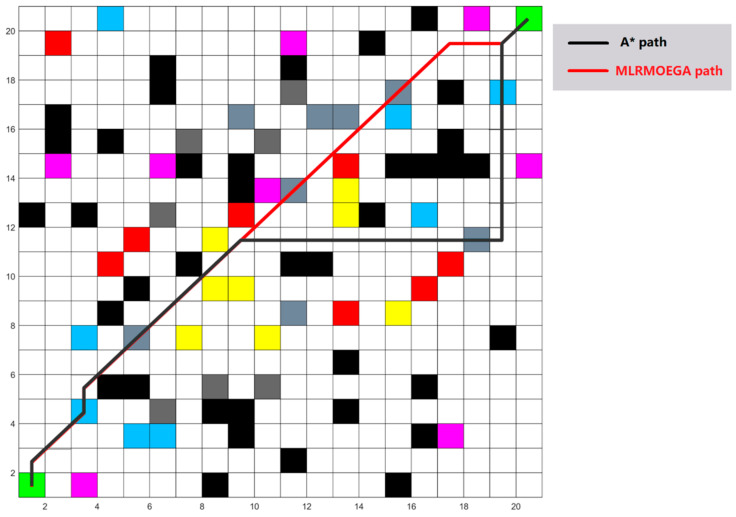
The test 20 × 20 grid map and paths.

One of the tests for the 20 × 20 grid map and paths generated by the two algorithms are shown in Figure 18, and the value of the objective function is shown in Table 9.

As can be seen from the results, under the equal weight of the three objective functions, the path generated by the multi-objective A* algorithm selected a different grid from MLRMOEGA path when the multi-objective A* algorithm was searching for the next grid at one grid, resulting in the final data being inferior to the MLRMOEGA path. Due to MLRMOEGA’s better global search ability, the objective function values of the path generated by MLRMOEGA are better.

The values of the synthetic objective function of other ten tests are shown in Table 10. As can be seen from the data in this table, due to its better global search ability, the objective function values of the paths generated by MLRMOEGA is less than that of the path generated by the multi-objective A* algorithm in most cases, and the optimization effect of MLRMOEGA is significantly better than that of the multi-objective A* algorithm (the *p*-value of the data in Table 10 is 0.004886).

#### 5.4.3. Comparison with Multi-Objective ACO

In this section, we compared the MLRMOEGA with a kind of a multi-objective ACO algorithm. The ant colony optimization (ACO) algorithm is a stochastic-based optimization technique that replicates the behavior of real ants when searching for food. The ants move along the same path by following one another. This is because every ant leaves a chemical substance called pheromone while moving on the path. The other ants sense the intensity of the pheromone and follow the path with a higher concentration of pheromone. This is their tactic to find an optimized path. On their back tour, the ants sense the pheromone intensity and choose the path with a higher concentration of pheromone [23].

**Figure 19:**
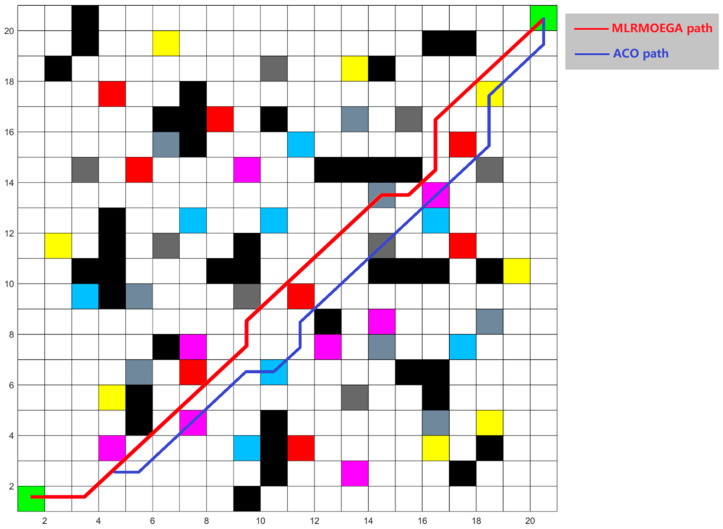
The test 20 × 20 grid map and paths.

This multi-objective ACO algorithm has the same four optimization objectives as the proposed algorithm, and we set these four objective function weights to 0.25 to consider an even performance of path planning. The test is performed on random 20 × 20 grids maps, which were generated in the same way as in Section 5.4.2. One of the tests for the 20 × 20 grid map and paths generated by the two algorithms are shown in Figure 19, and the value of the objective function is shown in Table 11.

As can be seen from the results, under the equal weight of the four objective functions, the multi-objective ACO algorithm generated a different path from MLRMOEGA. Because of the positive feedback of pheromone, the ant colony algorithm is easy to fall into local optimal solution in the process of approaching the optimal solution. With better global search ability, the objective function values of the path generated by MLRMOEGA are significantly lower.

The values of synthetic objective function of the other ten tests are shown in Table 12. As can be seen from the data in this table, due to its better global search ability, the objective function values of the paths generated by MLRMOEGA is less than that of the path generated by the multi-objective ACO algorithm, and the optimization effect of MLRMOEGA is significantly better than that of multi-objective ACO algorithm (the *p*-value of the data in Table 12 is 0.008118).

#### 5.4.4. Performance Test in Simulated Environment

**Figure 20:**
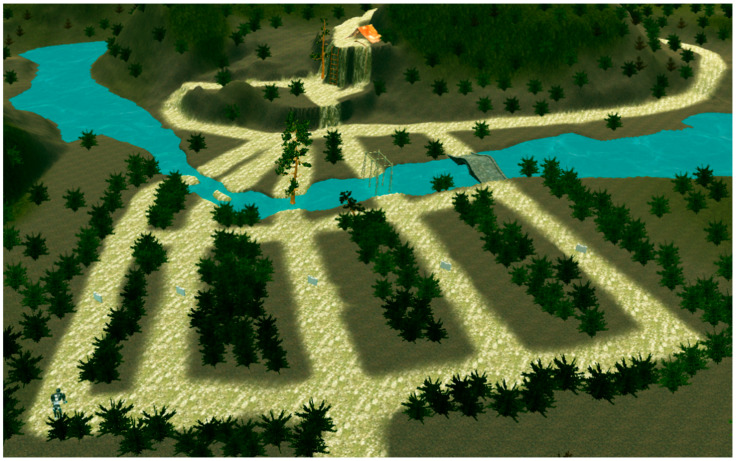
The simulated environment.

**Figure 21:**
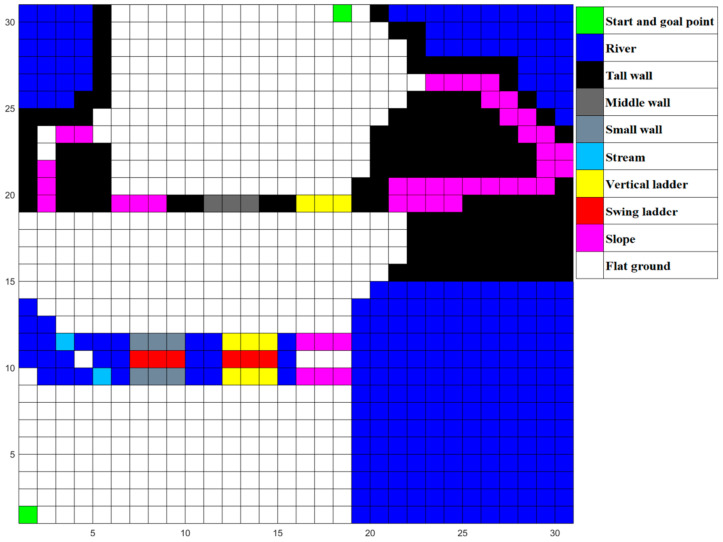
The abstracted 30 × 30 grid map of the simulated environment.

In order to test the performance of the proposed algorithm in the real world, we design a simulation environment close to the real situation as shown in Figure 20, in which the MLR had to choose various paths to cross the river, such as jumping over the stream, swinging on a ladder, walking over a bridge, and then climbing up slopes or walls to reach the destination. We abstract the simulated environment into a 30 × 30 grid map as shown in Figure 21. Then, we run MLRMOEGA and SGA in this map to compare the performance of the proposed algorithm.

**Figure 22:**
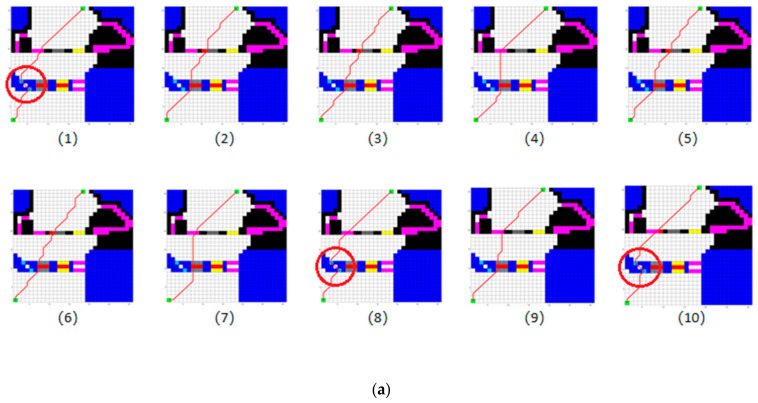

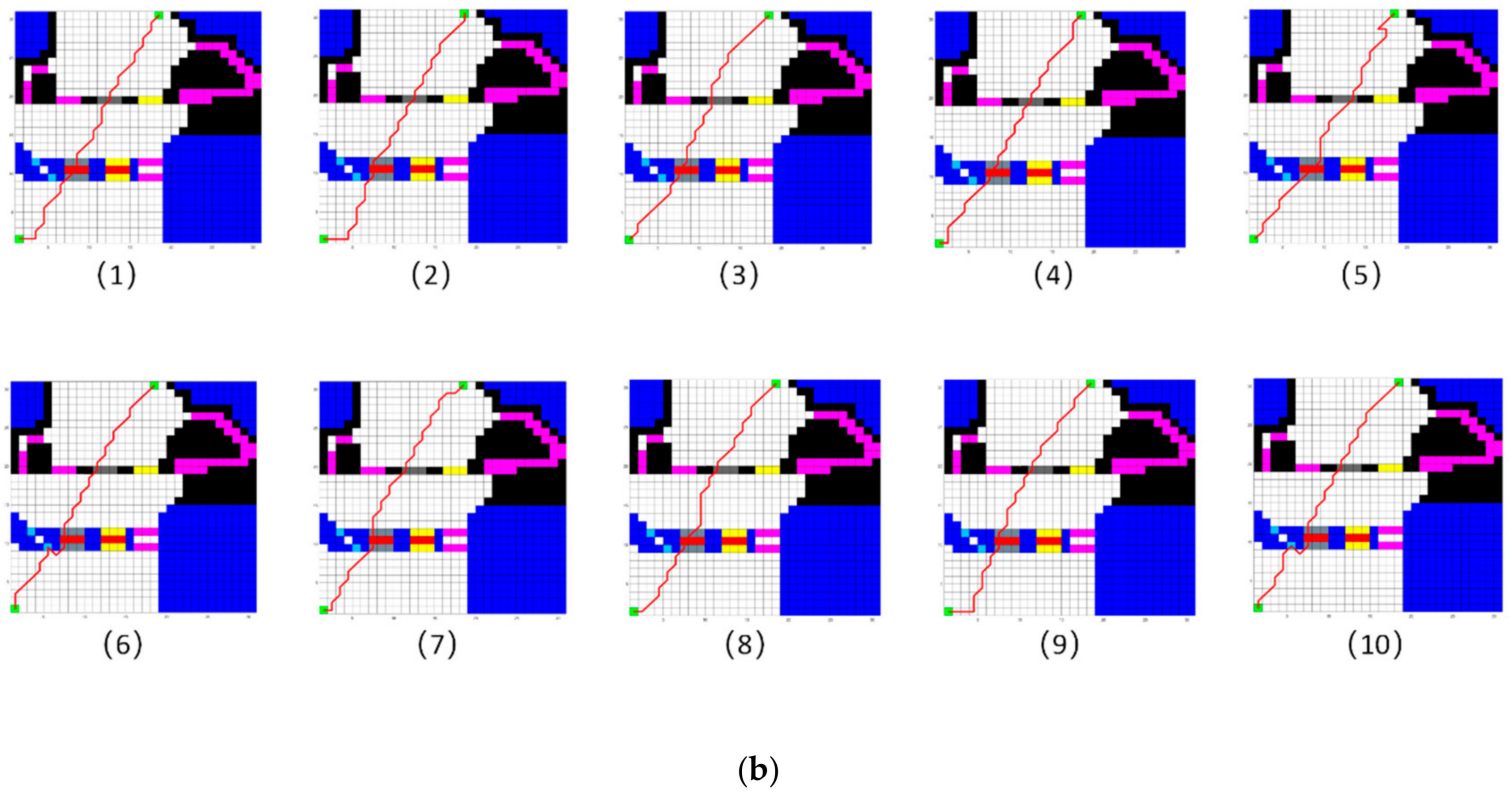
The paths of 30 × 30 grid map generated by: (**a**) MLRMOEGA; (**b**) SGA.

The design parameters of MLRMOEGA and SGA are shown in Table 6. The results of the 30 × 30 grid map are shown in. To clarify, Figure 22 shows the paths generated by different algorithms, obviously, under the same design parameters. Compared with SGA, the MLRMOEGA has better global search performance for better paths through narrow regions, for example, the stream region in the testing environment. In the 10-times test, the SGA did not generate a path passing through the stream region while the MLRMOEGA generated 3 paths passing through the stream region as shown in Figure 22a circled in red, and the paths passing through the stream region have a better synthetic objective function value (the *p*-value of the data in Table 13 is 0.00009459).

#### 5.4.5. Multi-Objective Optimization Performance Test

We test the multi-objective optimization performance of the proposed algorithm by changing the weight of the optimization target, and the test environment is the abstracted simulated environment shown in Figure 20.

The test results are shown in Table 14, the red text indicates the value of the objective function with the weight of one which is emphasized by the decision makers. From the result, we can see that the proposed algorithm can realize the intention of decision makers and effectively optimize the value of one certain objective function emphasized by the decision makers, and when the four optimization objectives are considered equally, the path generated by the proposed algorithm is relatively balanced, which proves the multi-objective optimization effectiveness of the proposed algorithm.

## 6. Conclusions

This paper proposed a multi-objective path planning algorithm for a multi-locomotion robot based on a genetic algorithm with elitist strategy. First, we determine four optimization objectives: power consumption, time consumption, falling risk, and smoothness, then set their objective functions and evaluation functions. Then, to solve the problem of premature convergence of SGA, we propose two operators: a map analysis operator and a population diversity expansion operator, to improve the population diversity in the algorithm process. We run the proposed algorithm in 30 × 30 grids testing environment, and the optimal design parameters are determined by balancing the execution time and the synthetic objective function value. After obtaining the optimal design parameters, we test its performance and compare the proposed algorithm in multiple environments with SGA. The results show that the proposed algorithm can effectively improve the global search ability and convergence of SGA. We also compare the proposed algorithm with a multi-objective A* algorithm. We run these two algorithms in a random 20 × 20 grid map; the results show that the synthetic objective function value of the MLRMOEGA path is better than that of the multi-objective A* algorithm path under equal weight due to the better global search ability of MLRMOEGA. Then, we test the performance of the proposed algorithm in a simulation environment which is close to the real field environment; the results show that the global search ability and optimization ability of MLRMOEGA is better than that of SGA. In addition, we test the multi-objective optimization performance under alternative weights, and we find that the output path results can effectively optimize the value of objective functions that the decision maker emphasizes.

According to the results above, the MLRMOEGA proposed in this paper can be effectively applied to MLR tasks, such as pipeline maintenance, medical care by micro-robots, cargo transporting, and terrain exploration by humanoid robots.

## Figures and Tables

**Table 1:** The assumed MLR data.

Locomotion Mode	Power Consumption	Speed	Falling Risk
Bipedal Walking	5	0.5	0.035
Quadruped Walking	10	0.3	0.02
Upward Climb	15	0.15	0.01
Swing	20	0.2	0.0015
High Jump	16	0.6	0.04
Climb Over	25	0.1	0.03
Long Jump	16	0.55	0.045

**Table 2:** Parameters of SGA and MLRMOEGA.

Parameters	SGA	MLRMOEGA
Initial Population Quantity	300	300
Iteration Limit	150	150
Crossover Probability	0.95	0.95
Mutation Probability	0.2	0.2
Unit Grid Length	1	1
Vertical Ladder Height	2	2
*cw*	-	0
*tw*	-	0
*rw*	-	1
*aw*	-	0

**Table 3:** Results of Artificial Potential Field Theory Experiment (with 20 × 20 grid map).

Algorithm	Falling Risk	Average Value	Algorithm	Falling Risk	Average Value
MLRMOEGA	0.7442	0.7498 ± 0.0186	SGA	0.8194	0.7952 ± 0.0248
	0.7303			0.7826	
	0.7409			0.8053	
	0.7303			0.7326	
	0.7908			0.8133	
	0.7438			0.8053	
	0.7637			0.7788	
	0.7618			0.8194	
	0.7623			0.7901	
	0.7303			0.8053	

**Table 4:** Results of Artificial Potential Field Theory Experiment (with 30 × 30 grid map).

Algorithm	Falling Risk	Average Value	Algorithm	Falling Risk	Average Value
MLRMOEGA	0.7303	0.7176 ± 0.0073	SGA	0.7137	0.7760 ± 0.0451
	0.70868			0.71719	
	0.70868			0.75059	
	0.70868			0.76285	
	0.71458			0.78128	
	0.71458			0.78128	
	0.72168			0.78128	
	0.72168			0.79262	
	0.72168			0.79632	
	0.72604			0.88266	

**Table 5:** Other parameters of the proposed algorithm.

Parameters	Value
Crossover Probability	0.95
Mutation Probability	0.2
Unit Gird Length	1
Vertical Ladder Height	2
*cw*	0.25
*tw*	0.25
*rw*	0.25
*aw*	0.25

**Table 6:** The parameters of SGA and MLRMOEGA.

Parameters	SGA	MLRMOEGA
Crossover Probability	0.95	0.95
Mutation Probability	0.2	0.2
Iteration Limit	130	130
Initial Population Quantity	200	200

**Table 7:** The results of the test (20 × 20 grid map).

Algorithm	MLRMOEGA	SGA
Weight	0.25; 0.25; 0.25; 0.25	0.25; 0.25; 0.25; 0.25
1st	62.1595	83.4842
2nd	81.7038	114.4333
3rd	64.9642	85.7737
4th	62.7269	110.1055
5th	81.7038	83.2888
6th	63.0997	75.1382
7th	73.0977	118.8659
8th	65.7390	114.5052
9th	81.7038	86.3457
10th	104.9453	83.9620
Average Value	74.1844 ± 12.9310	95.5902 ± 15.8066

**Table 8:** The results of 5 other tests (20 × 20 grid map).

Map	Algorithm	MLRMOEGA	SGA
	Weight	0.25; 0.25; 0.25; 0.25	0.25; 0.25; 0.25; 0.25
Map.1	1st	52.7883	60.9763
	2nd	52.7883	62.0930
	3rd	52.7889	53.3597
	4th	53.8005	63.6244
	5th	60.3864	58.0548
	6th	52.7889	52.9650
	7th	52.7883	52.7883
	8th	52.7883	60.9776
	9th	60.1569	55.8255
	10th	52.7883	61.7462
	Average Value	54.3863 ± 2.9583	58.2402 ± 3.9792
Map.2	1st	60.5829	79.3548
	2nd	56.2152	95.4599
	3rd	58.3483	95.6548
	4th	57.6904	68.3099
	5th	54.1988	68.3099
	6th	56.2152	85.2334
	7th	55.6137	97.6310
	8th	64.8534	65.0661
	9th	64.8534	66.8496
	10th	56.2152	65.0661
	Average Value	58.4786 ± 3.5810	78.6936 ± 13.0306
Map.3	1st	63.309	73.35
	2nd	63.5583	89.7417
	3rd	62.688	75.3931
	4th	63.7569	105.3384
	5th	63.309	88.2386
	6th	63.3095	89.5278
	7th	63.7569	87.9132
	8th	60.7829	86.7099
	9th	64.3559	87.9132
	10th	63.7569	87.9132
	Average Value	63.2583 ± 0.9220	87.2039 ± 8.2155
Map.4	1st	73.1337	103.478
	2nd	73.1353	106.5394
	3rd	73.1353	111.6177
	4th	73.1337	106.3428
	5th	81.6376	75.3689
	6th	73.1337	75.7625
	7th	73.1337	92.1121
	8th	73.1353	104.9162
	9th	73.1353	93.1986
	10th	69.2510	89.6151
	Average Value	73.5965 ± 2.9198	95.8951 ± 12.2319
Map.5	1st	52.5312	55.3959
	2nd	52.5312	65.2409
	3rd	52.5312	60.7812
	4th	52.5312	56.6374
	5th	52.3362	52.9624
	6th	52.3362	54.7224
	7th	52.3362	52.9624
	8th	52.3362	54.7224
	9th	52.5655	61.0621
	10th	52.7605	53.1563
	Average Value	52.4796 ± 0.1339	56.7643 ± 3.9826

**Table 9:** Results of test compared with multi-objective A* algorithm.

Algorithm	MLRMOEGA	Multi-Objective A* Algorithm
Weight	0.3; 0.3; 0.3; 0	0.3; 0.3; 0.3
Power Consumption	142.4	155.6
Time Consumption	48.6284	54.8182
Path Falling Risk	0.7756	0.8356
The synthetic objective function	57.5412	63.3762

**Table 10:** The values of synthetic objective function compared with multi-objective A* algorithm.

Algorithm	MLRMOEGA	Multi-Objective A* Algorithm
Weight	0.3; 0.3; 0.3; 0	0.3; 0.3; 0.3
Map 1	59.1161	90.9409
Map 2	60.8907	66.9772
Map 3	60.3342	77.0933
Map 4	58.9753	49.1492
Map 5	58.9010	64.8626
Map 6	59.5881	76.4095
Map 7	57.6954	68.5558
Map 8	58.9312	91.6395
Map 9	59.9457	76.3552
Map 10	59.8486	79.3438

**Table 11:** Results of test compared with multi-objective ACO algorithm.

Algorithm	MLRMOEGA	Multi-Objective ACO Algorithm
Weight	0.25; 0.25; 0.25; 0.25	0.25; 0.25; 0.25; 0.25
Power Consumption	142.9371	222.4558
Time Consumption	55.7548	82.0036
Path Falling Risk	0.8036	0.9371
Path Smoothness	5.4978	11.7810
The synthetic objective function	51.2483	79.2944

**Table 12:** The values of synthetic objective function compared with multi-objective ACO algorithm.

Algorithm	MLRMOEGA	Multi-Objective ACO Algorithm
Weight	0.25; 0.25; 0.25; 0.25	0.25; 0.25; 0.25; 0.25
Map 1	51.1325	108.3110
Map 2	50.0654	195.0888
Map 3	48.8652	81.6816
Map 4	49.5720	73.6517
Map 5	50.4595	107.5477
Map 6	50.4761	67.1134
Map 7	50.8855	67.2223
Map 8	48.8173	68.8862
Map 9	49.8645	79.6593
Map 10	48.0516	68.6640

**Table 13:** The results of test (30 × 30 grid map).

Algorithm	MLRMOEGA	SGA
Weight	0.25; 0.25; 0.25; 0.25	0.25; 0.25; 0.25; 0.25
1st	71.2978	84.6640
2nd	80.0754	85.9012
3rd	81.254	83.6812
4th	69.9468	85.6848
5th	80.6644	86.3729
6th	81.2546	87.3921
7th	70.5163	85.2559
8th	71.3646	85.1268
9th	70.5464	85.7125
10th	71.7590	87.3930
Average Value	74.8679 ± 4.8867	85.7184 ± 1.0906

**Table 14:** Results of Multi-Objective Optimization Performance Test.

Algorithm	MLRMOEGA				
Weights	1; 0; 0; 0	0; 1; 0; 0	0; 0; 1; 0	0; 0; 0; 1	0.25; 0.25; 0.25; 0.25
Power Consumption	200.7351	201.9777	227.3082	201.8792	207.7371
Time Consumption	74.0966	73.6447	85.0833	73.9689	76.312
Path Falling Risk	0.9357	0.9374	0.7217	0.7975	0.8412
Path Smoothness	10.9956	10.2102	8.6394	1.5708	7.0686
